# Green genes from blue greens: challenges and solutions to unlocking the potential of cyanobacteria in drug discovery

**DOI:** 10.1039/d5np00016e

**Published:** 2025-07-15

**Authors:** Benjamin Philmus, Nicole E. Avalon, Yousong Ding, Drew T. Doering, Alessandra S. Eustáquio, William H. Gerwick, Hendrik Luesch, Jimmy Orjala, Shaz Sutherland, Arnaud Taton, Daniel Udwary

**Affiliations:** a Department of Pharmaceutical Sciences, Oregon State University Corvallis OR 97333 USA benjamin.philmus@oregonstate.edu; b Department of Pharmaceutical Sciences, School of Pharmacy & Pharmaceutical Sciences, University of California, Irvine Irvine CA 92617 USA; c Department of Medicinal Chemistry, Center for Natural Products, Drug Discovery and Development, University of Florida Gainesville FL 32610 USA; d U.S. Department of Energy, Joint Genome Institute, Lawrence Berkeley National Laboratory One Cyclotron Road Berkeley CA 94720 USA; e Department of Pharmaceutical Sciences, Retzky College of Pharmacy, University of Illinois, Chicago Chicago IL 60612 USA; f Center for Marine Biotechnology and Biomedicine, Scripps Institution of Oceanography, and Skaggs School of Pharmacy and Pharmaceutical Sciences, University of California San Diego La Jolla CA 92092 USA; g Program in Cancer and Stem Cell Biology, Duke-NUS Medical School Singapore 169857 Singapore; h School of Biological Sciences, University of California, San Diego La Jolla CA 92093 USA

## Abstract

Cyanobacteria are prolific producers of biologically active compounds that are important in influencing ecology, behavior of interacting organisms, and as leads in drug discovery efforts. Here we discuss the challenges faced by all natural product researchers, especially those that focus on cyanobacteria, and then describe progress that has been made in these areas. We also propose some solutions, paths forward, and thoughts for consideration on these challenges.

## Abstract 2

Cyanobacteria are prolific producers of bioactive natural products. In this article, we outline some challenges faced in exploiting cyanobacteria for drug discovery, current solutions, and pathways forward to fully explore them for drug discovery.

## Graphical abstract

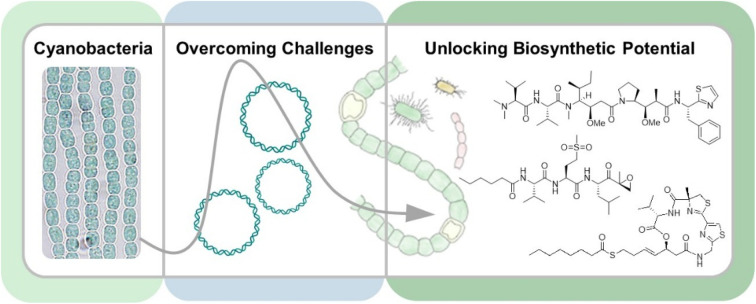

## 1. Background

Cyanobacteria, also known as ‘blue-green algae’, emerged approximately 3 billion years ago and have since developed a remarkable array of metabolic traits through extensive evolutionary adaptation. These include oxygenic photosynthesis, nitrogen fixation, UV and desiccation tolerance, and the production of a diverse repertoire of secondary metabolites. Notably, their ability to perform oxygenic photosynthesis is credited with transforming Earth's atmosphere into its current oxygen-rich state and continues to have profound implications for future climate scenarios. Since the 1970s, cyanobacteria have been acknowledged as an exceptional source of bioactive and structurally diverse secondary metabolites. More recently, cyanobacteria have been recognized as a carbon-negative chassis for synthetic biology applications, including production of biofuels, sunscreens, and drug leads.^1^

Cyanobacterial natural products play significant environmental and ecological roles.^2–4^ In aquatic environments ranging from freshwater to marine, species such as *Microcystis* and *Nodularia* produce cyclic peptides, including microcystins and nodularins, which exhibit potent protein phosphatase inhibition.^5,6^ When ingested, these compounds are highly toxic to the liver of mammalian species. These toxigenic cyanobacteria can grow to high cell densities and lead to harmful cyanobacterial blooms, posing serious ecological and public health challenges.^7^

Cyanobacteria have also been pivotal in Earth's evolutionary history, predating the presence of atmospheric oxygen and the protective ozone layer that shields against UV-A and UV-B radiation.^8^ In response to these harsh conditions, they evolved the ability to produce natural products that protect against UV damage. For example, the mycosporine-like amino acids (MAAs), such as shinorine, are small molecules featuring an aminocyclohexenone ring that provide effective protection against UV-B radiation.^9,10^ Similarly, scytonemin, an indole alkaloid dimer produced by many cyanobacterial species, offers protection against UV-A wavelengths. These compounds highlight the adaptive versatility of cyanobacteria in diverse and challenging environments.^11^

Cyanobacterial metabolites exhibit distinctive features, such as nitrogen-rich frameworks, covalent incorporation of halogen atoms, and hybrid structures integrating components from different biosynthetic pathways.^12^ Biologically, cyanobacterial secondary metabolites target a variety of molecular processes, including proteases, ion channels, tubulin, actin, and other critical cellular features of potential eukaryotic competitors. This specificity has particularly positioned cyanobacterial compounds as promising candidates for anticancer and antiproliferative therapies.^13^

Prominent examples of marine cyanobacterial natural products with anticancer or potential anticancer activity include dolastatin 10,^14^ curacin A,^15^ and apratoxin A,^16^ each notable for their architectural complexity and unique mechanisms of action. These compounds derive their distinctive chemical structures from the integrated activities of nonribosomal peptide synthetases (NRPS) and polyketide synthases (PKS), yet their structural diversity results in markedly different biochemical properties.

**Fig. 1:**
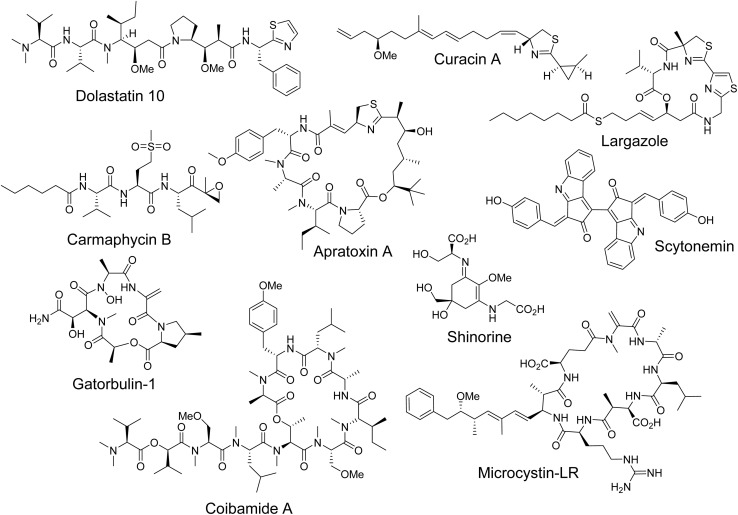
Cyanobacterial compounds of note. These compounds represent important drug leads (dolastatin 10, apratoxin A, largazole, coibamide A, carmaphycin B) or compounds with importance in the environment (microcystin-LR, shinorine, scytonemin, curacin A) isolated from freshwater, terrestrial, and marine cyanobacteria.

The potent microtubule depolymerizing agent dolastatin 10, a modified linear peptide–polyketide produced by the marine cyanobacterium *Caldora penicillata*, served as the starting point for the development of monomethyl auristatin E (MMAE), the cytotoxic payload of five currently approved antibody–drug conjugates (ADCs). The success of brentuximab vedotin, the first MMAE ADC for the treatment of Hodgkins lymphoma and anaplastic large cell lymphoma in 2011, and now a blockbuster drug with over $1B in annual sales, contributed to the ADC revolution and validated this enabling technology as a therapeutic modality.^17^ Dolastatin 10 (Fig. 1) is a linear hexapeptide featuring two amino acids that are PKS-extended by two carbons each *via* malonyl-CoA units. The C-terminal cysteine is cyclized and oxidatively decarboxylated to form a thiazole ring. Mechanistically, dolastatin 10 and its analogs inhibit microtubule assembly by binding to the vinca alkaloid site on the β-tubulin subunit, disrupting cellular division. Curacin A (Fig. 1), another structurally intriguing molecule, combines PKS-derived malonyl extensions with a single cysteine unit, producing a distinctive combination of cyclopropyl and thiazoline rings. As a potent antimitotic agent, curacin A binds to the colchicine site of microtubules, interfering with their dynamic behavior essential for mitosis. The gatorbulins (Fig. 1),^18^ modified cyclodepsipeptides targeting a new pharmacological site of tubulin, represent the most recent scaffold and mechanism that modulate tubulin dynamics.

Largazole (Fig. 1) is among the most potent histone deacetylase (HDAC) inhibitors discovered so far and has selectivity for class I isoforms, rivaling the approved drug romidepsin.^19^ A largazole analogue, bocodepsin, reached Phase I clinical trials. Carmaphycins (Fig. 1), linear modified peptides, are potent proteasome inhibitors and serve as a template for the development of organism-specific inhibitors.^20^ While the proteasome is a validated target for cancer, synthetic analogues of the carmaphycins possess selectivity for the parasite proteasome and are now in development to treat tropical parasite infections, including trypanosomiasis. The macrocyclic polyketide–peptide hybrids apratoxins and coibamides (Fig. 1) have been shown to perturb cotranslational translocation by targeting the translocon Sec61 in the endoplasmic reticulum, representing a new mechanism for inhibiting protein maturation.^21,22^ Elucidating the novel mechanism of these two agents has opened up opportunities for development for different indications linked to Sec61. A biosynthetic hybrid of three different moieties, a peptide–polyketide–glycoside termed iezoside was found to be a sarco/endoplasmic reticulum Ca^2+^ ATPase pump (SERCA) inhibitor, linking an unusual structure to function.^23^ Functionally, cyanobacterial natural products display a wide range of biological activities against diverse molecular targets. Synthetic structural modifications of their unique structures can lead to improved drug-like properties.

The remarkable structural diversity of cyanobacterial natural products stems from the unique reactivity and specificity of their biosynthetic enzymes. Over the past three decades, significant progress has been made in understanding the function of some of these enzymes, the pathways they catalyze, and the gene clusters that serve as the ultimate repository of this biosynthetic information.^24–26^ A natural extension of these discoveries has been the effort to harness and transfer this biosynthetic potential into other microbial systems that are more amenable to cultivation and genetic engineering. A summary of these efforts can be found in recent reviews by Dhakal *et al.*^27^ and Baunach *et al.*^28^ These efforts include the use of various traditional genetically tractable hosts such as *E. coli*, various Actinobacteria, and yeast as well as various model cyanobacterial species. The use of a cyanobacterial chassis allows for the environmentally friendly production of these high-value chemicals, as cyanobacteria are considered a sustainable platform for biotechnological applications due to their photoautotrophic and nitrogen-fixing capacities. But some technical barriers in working with cyanobacteria include low titers of their natural products (typically 0.1–0.2% dry weight), slow growth rates, and difficulty removing associated bacteria,^29,30^ which has led to them being underrepresented in drug discovery efforts.

This perspective aims to provide an overview of recent advancements in the use of cyanobacterial natural product pathway activation in native producers and capture, harness and expression of natural product BGCs in heterologous hosts, challenges faced by all researchers involved in natural product research, and issues specifically facing researchers working on cyanobacterial natural product pathways, while highlighting critical challenges that continue to impede progress in this promising area.

## 2. Challenges that impact all natural product researchers

### 2.1. General challenge #1: effective prioritization of BGCs

Previous research has identified up to 16 984 gene cluster families (GCFs) in 16 004 complete bacterial genomes with the majority (97%) being considered orphan biosynthetic gene clusters (BGCs).^31^ Hence, prioritization of BGCs to pursue for in-depth studies becomes essential. For example, assuming 20 kb for each cluster and a price of commercial synthesis of $0.09 per base, the cost of synthesizing one BGC from each GCF would be $30 571 200. This is without considering the salary of researchers to assemble the synthesized fragments into BGCs, transformation into appropriate heterologous production hosts, and chemical analysis. Similarly, cultivation of 16 004 bacterial strains in 3–4 media followed by chemical extraction and analysis would be an enormous undertaking. It is apparent that the prioritization of BGCs to work on is essential in order to maximize research efforts to find new chemistry and biochemistry, and to ensure the highest return on investment of research funding. To this end, multiple research groups have been engaged in orthogonal efforts to prioritize BGCs in different bacteria, which are briefly reviewed below. Prioritization of BGCs for expression is typically guided by one of the following goals:

(1) linking a compound of interest to a putative BGC, (2) exploring BGCs based on enzymatic novelty, (3) identifying specific enzymes or enzyme groups that would result in structural novelty, or (4) assessing bioactivity based on accessory genes and, (5) artificial intelligence (AI)-based approaches.

#### 2.1.1 Linking a compound of interest to a putative BGC

Most current strategies for linking compounds to their BGCs are heavily reliant on structure-based predictions arising from the BGC. However, an innate limitation is the need for well-characterized biosynthetic enzymology and substrate specificity. Additionally, while co-linearity can guide structural predictions in NRPS and type I-PKS compound classes, the enzymology of several compound classes such as alkaloids and terpenes currently lack the characterization required to directly predict structures from genes. These limitations can be addressed by both accelerating structure elucidation of natural products and by improving direct BGC-to-structure predictions. Computer-assisted structure elucidation (CASE) systems,^32^ AI strategies (as discussed in the next section), and approaches using advanced X-ray diffraction with CryoEM^33^ or microcrystalline sponges^34^ are advancing rapidly to facilitate structure determination. There is also an increasing push for the deposition of multi-omics data sets into public repositories, which aids in correlating genes, enzymes and structures.^35,36^ For example, NPAtlas^37^ currently contains 2137 cyanobacterial structures and CyanoMetDB (version 3) contains 3084 compounds;^38,39^ the Secondary Metabolite Collaboratory (SMC)^40^ currently contains over 4200 cyanobacterial genomes and 32 000 BGCs, while MIBiG only contains 146 experimentally verified BGCs linked to cyanobacterial compounds.^41^ Cross-referencing of biosynthetic repositories and compound-based repositories is underway and can be linked to omics datasets deposited in established databases.^42^ These linked datasets can be analyzed to improve the knowledge surrounding BGC-to-structure relationships and are critical to advancing the field. Tools such as NPLinker,^43^ IsoAnalyst,^44^ NPOmix,^45^ and strategies such as inverse stable isotopic labeling^46,47^ all use muti-omics techniques developed to address this gap. As technological and computational advances are made, natural product scientists can explore the unknown biosynthetic spaces through these emerging strategies and techniques. Although none of these tools are exclusive to cyanobacterial natural product discovery, all can be applied.

#### 2.1.2 Structural novelty

One fruitful strategy that has been used to prioritize BGCs is to group them in gene cluster families (GCFs).^48^ Although BGC predictions are often rule-based, by selecting BGCs that are most unique, the likelihood of structural novelty increases. As multi-omic datasets become increasingly available along with better BGC-to-structure predictions, it is possible that the emphasis for novelty can be placed on predicted structures rather than solely on BGC similarity or dissimilarity. This is important, as convergent evolution allows for multiple pathways to produce the same structural feature(s), a situation that could be captured by predicted structure-based strategies rather than genomic content alone. This can also allow for the prediction of structures arising from difficult to characterize classes.

#### 2.1.3 Enzymatic novelty or exploration

Enzyme centric strategies can also be employed to prioritize BGCs encoding novel chemical entities. Biosynthetic queries for unusual genes, uncommon pathways, and unique moieties can identify strains with these features, and then these strains can be cultivated and queried for the targeted natural products. This type of searching can be performed by BLAST analysis^49,50^ on sequenced genomes or by PCR of strain collections that have not yet been sequenced.^51^ Biosynthetic mining can be coupled with isotope labeling to facilitate the identification of compounds of interest.^52^ However, this approach relies on prior knowledge of desired chemical features as well as of the enzymes responsible for key biochemical transformations.

#### 2.1.4 Bioactivity

Another approach of BGC prioritization involves targeting bioactivity by focusing on accessory genes such as those conferring antibiotic resistance, genes involved in transport, or other genes of interest. The prevalence of resistance markers in cyanobacteria are understudied, but some studies suggest that up to 90% of cyanobacteria have genes conferring resistance to multiple antibiotics.^53^ The Antibiotic Resistant Target Seeker (ARTS) identifies resistance and housekeeping genes in proximity to BGCs, gene duplication, and evidence of horizontal gene transfer to aid in the prioritization of BGCs that are likely responsible for antibiotic production.^54^ Machine learning has also been utilized to predict which BGCs encode bioactive natural products based on sub-structures predicted to be in the synthesized natural product.^55–57^ Additional experimental approaches in development combine structure predictions from gene clusters (*e.g.*, antiSMASH, PRISM) with predictions of biological activity from the predicted structures (PECAN).^58^ Tools such as these that enhance our ability to identify unique, niche, or substructures for desired targets from BGC enzymology will enable more efficient prioritization of genomic data for heterologous expression.

#### 2.1.5 The use of AI to address the challenge of prioritization

Computational strategies that provide analytical and bioinformatic insights into the relationships between genes, products, and biological properties can be further enhanced by AI. In a recent review, the current utility of AI in natural product discovery has been comprehensively outlined and we refer our readers to this review.^59^

One of the bottlenecks in linking BGCs to their encoded natural products is the innate need for unambiguous structure elucidation of a natural product to then allow for retrobiosynthesis and linking of a congruent BGC. NMR-based approaches such as SMART2.0,^60^ SMART-Miner,^61^ and DeepSAT^62^ are accelerating structure elucidation through AI recognition of HSQC spectra as interpreted in the algorithm as either multidimensional vectors or Morgan Fingerprints and then matched with structural features of known NPs. The output provides the structures of known compounds with similar structural features, providing a jumpstart to the elucidation process. Mass spectrometry-based approaches such as Spec2Vec,^63^ MS2DeepScore,^64^ and COSMIC^65^ that mine the metabolome enhance fragment identification and compound and analogue annotation, thus also accelerating the structure elucidation process. Other tools such as NPOMix^45^ utilize AI and ML to link BGCs to mass spectral data. These tools are quite dependent upon paired, high-quality training sets, which are often pulled from public repositories, though at the current time are limited in number. Repositories and analysis tools designed specifically for paired datasets such as NPLinker,^43^ GraphOmics,^66^ and Anvi'o^67^ exist and are expanding to meet the growing needs within the community.

Since the final goal for many NP discovery efforts is targeting human disease, predicting bioactivity from structure and/or BGCs is an appealing application of AI and an important consideration for prioritization of BGCs for heterologous expression. Building upon the ideas for targeting resistance, regulation, and key evolutionary genes, AI can be used to identify new markers of resistance, identify key genes or chemical substructures associated with bioactivity, and apply this knowledge to prioritization schemes for BGCs producing biologically active molecules. For example, chemical structures have been used to predict bioactivity in tools such as PECAN,^58^ while nucleotide and amino acid sequences from BGCs have also been used to predict bioactivities,^57^ allowing for another level of BGC prioritization for expression, testing, and potentially development of natural products. Future advancements of new AI and ML models would be beneficial to accelerate structure determination, and linking BGCs with compound production and their resulting biological properties.

### 2.2. General challenge #2: many natural product pathways are large and complex

Natural product BGCs within bacterial genomes range in size from 5 kbp to 200 kbp with the total number of discrete BGCs correlating with genome size.^48^ In addition, BGC size and the number of genes often relates directly to the structural complexity of the associated natural product. However, cloning and successful expression of large BGCs takes both financial and temporal resources.

One of the issues facing synthetic biologists is the lack of large-scale studies that detail successes and failures of natural product BGCs, particularly the failures. As is typical in science, only the successes are reported in publications leaving the failures hidden in laboratory notebooks. As Samuel Smiles wrote, “We learn wisdom from failure much more than from success. We often discover what will do, by finding out what will not do; and probably he who never made a mistake never made a discovery”.^68^ As noted by Kadjo and Eustáquio,^69^ success rates for the four large scale heterologous expression studies reported range between 11–32% suggesting that optimization of hosts, development of new hosts in addition to large scale studies, potentially collaborative across laboratories, would be beneficial to the community as a whole. In some of these large-scale studies both positive and negative results are reported to provide data so that guidelines can be applied to maximize success in heterologous expression experiments. These data can also be used to populate machine learning or artificial intelligence data sets for training.

The main bottlenecks in heterologous expression include building expression vectors, cultivating genetically engineered organisms and performing chemical extractions, compound isolation, and full structure elucidation. The automation of these steps could enhance workflows and throughput. One additional hurdle is the lack of institutional knowledge and training programs for using non-model organisms. While vectors and strains are frequently shared, the tips and tricks with non-model organisms are sometimes lost when a trainee leaves a lab. Additionally, depending on project priorities, the successes and failures of industry researchers may be left unpublished.

To overcome some of these barriers, the formation of consortiums to pool resources and results can amplify efficiency and promote success in heterologous expression. Funded centers where trainees could come and learn techniques, protocols, and interact with experts will facilitate the adoption of heterologous expression in more laboratories. Initial studies with both model and non-model organisms could focus on which clusters are successfully expressed including metadata such as original organism, BGC size, plasmid assembly methods, heterologous host, among others. This could be expanded in the future to include some of the nuances important in expression, such as regulators present in the BGC and the need for precursor pathways. The formation of Biofoundries by the Department of Energy is a promising beginning in this direction.

## 3. Challenges faced by natural products researchers focusing on cyanobacteria

### 3.1. Richness and diversity of cyanobacterial BGCs: reanalysis of high-quality genomes reveals the enormous potential and opportunity cyanobacteria provide

**Fig. 2:**
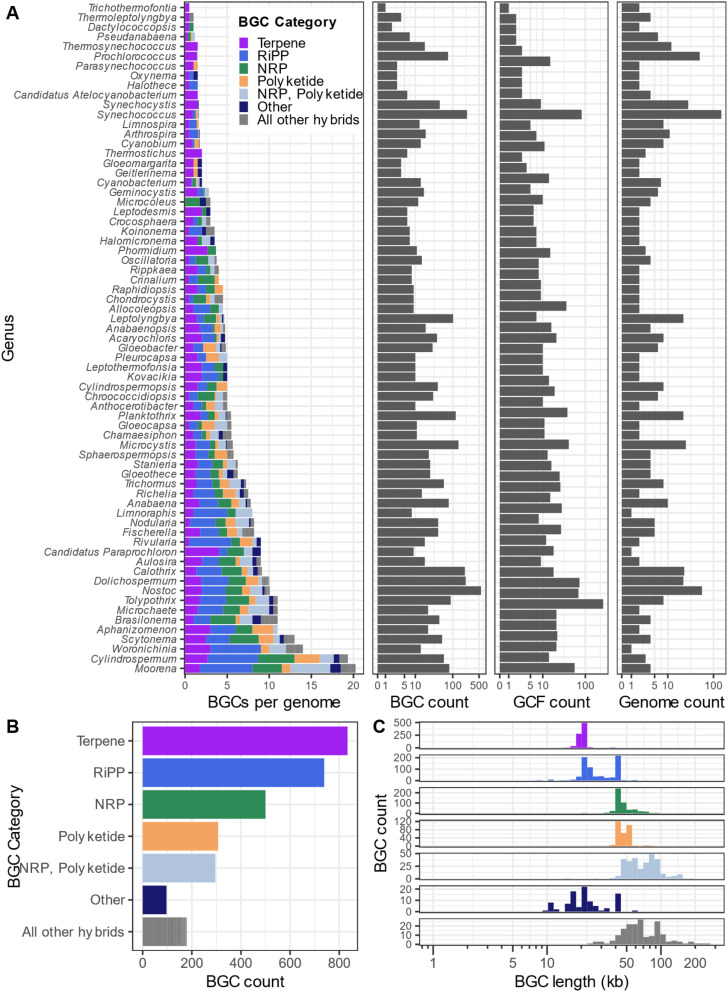
High-level survey of cyanobacterial BGCs from high-quality genomes found in SMC. (A) Number of BGCs per genome, total BGC count, gene cluster family (GCF) count (as determined by BiG-SLiCE, threshold = 0.4), and total genome count across high-quality genomes in cyanobacterial genera. Different categories of natural product BGCs are shown in different colors (as defined by AntiSMASH). (B) Total BGC counts found in high-quality cyanobacterial genomes, colored and separated by BGC category. (C) Distribution of BGC length (in kb) by BGC category. BGCs were annotated by AntiSMASH and subsequently clustered into GCFs using BiG-SLiCE.

We surveyed the Secondary Metabolite Collaboratory (SMC) to provide an overview of BGCs found in cyanobacterial genomes (Fig. 2). We identified 32 112 BGCs in 4090 cyanobacterial genomes (data accessed 2/26/2025). Since fragmented and incompletely-assembled genomes often have BGCs split across contigs, we filtered our dataset to genomes of “chromosome” or “complete” assembly quality, as listed on NCBI. After filtering, our dataset consisted of 2953 BGCs in 1881 GCFs across 326 cyanobacterial genomes (302 genomes did not have a BGC detected by AntiSMASH). Of these annotated BGCs, 834 are classified as terpene, 739 are classified as ribosomally synthesized and post-translationally modified peptides (RiPPs), 500 are classified as NRPSs, 307 are classified as PKS, 296 are classified as NRPS–PKS hybrids, 97 are classified as “Other” and 180 are classified as combinations of BGC categories. In contrast, MIBiG 4.0 (ref. 41) contains 146 linked natural product/BGC pairs from cyanobacteria demonstrating the potential of new compounds and pathways that remain to be discovered from cyanobacteria.

We calculated the distribution and average size of cyanobacterial BGCs (Fig. 2). The majority of NRPS and PKS BGCs are within the range of 45–60 kb, while the majority of NRPS–PKS hybrids are larger (50–110 kb), terpene and RiPP BGCs are generally smaller in size (*ca.* 20 kb). The majority of NRPS, PKS, and NRPS–PKS hybrid BGCs should be accessible by established methodologies developed for cloning large DNA fragments.^70^ As previously mentioned, 2142 compounds are annotated in NPAtlas as deriving from cyanobacteria whereas 22 833 BGCs (not including terpene BGCs) were identified in SMC from this phylum; therefore, a maximum of only 9.4% of the chemical diversity of cyanobacteria has been discovered to date. To our knowledge, no lanthipeptides have been isolated from cyanobacteria, except the prochlorosins, despite the presence of LanM homologs in multiple genomes, suggesting an untapped resource for the discovery of novel lanthipeptides. We note that the estimated terpene and RiPP BGC sizes of *ca.* 20 kb is most likely larger than the true lengths of these BGCs. These sizes are an artifact of the definition of BGC boundaries by AntiSMASH which identifies a biosynthetic core gene (*e.g.* terpene cyclase) and extends 10 kb in either direction and then examines the genomic DNA for additional core biosynthetic genes. This procedure most likely inflates the size of terpene and RiPP BGCs.

### 3.2. Cyanobacterial specific challenge #1: accessing NP biosynthesis by native producers

#### 3.2.1 Culture conditions and eliciting factors for NP biosynthesis

The relatively slow growth rate of cyanobacterial cultures is a limiting factor for the discovery and characterization of new NPs. However, the prevalence of cyanobacteria in diverse natural niches suggests that optimizing culture conditions can overcome some aspects of this limitation. For example, increased nitrogen (N) and phosphorus (P) levels together with higher summer temperatures are often associated with toxic blooms.^71^ Cyanobacterial growth is directly linked to the photosynthetic rate, which depends on light intensity, CO_2_ fixation, and temperature. As cultures grow, light penetration decreases due to self-shading, while high light intensities can cause photoinhibition and photobleaching; therefore, careful management of light intensities is important. Careful management of CO_2_ level is also essential, as it significantly impacts culture pH critical for growth. Both factors likely impact the synthesis of NPs. High-density (HD) cultivation seems to overcome these difficulties resulting in improved growth of several cyanobacterial strains and has led to faster growth and reprogrammed BGC expression in *N. punctiform*e PCC 73102.^72^

Other abiotic factors may also impact the regulatory networks governing NP biosynthesis. For example, the production of scytonemin and mycosporine-like amino acids (MAAs) is stimulated by UV and other stresses.^73^ The production of different jamaicamide analogs occurs at different times of the day,^74^ suggesting an alignment with the circadian clock or an effect of light conditions. Siderophores such as cyanochelins are stimulated by iron deprivation,^75^ while the eagle-killing toxin aetokthonotoxin (AETX) requires the accumulation of potassium bromide from a plant partner.^76^

Despite the advances described above, a vast knowledge gap exists for the roles of specialized metabolomes and their diverse inducing cues in comparison to the rich diversity of known compounds and identified cyanobacterial BGCs. Systematically evaluating culture conditions in conjunction with multi-omics approaches, integrating genomics, transcriptomics, proteomics, and metabolomics data, can provide a comprehensive understanding of how these factors influence growth and BGC expression. In addition, HD cultivation approaches could be deployed at different scales for NP research. Furthermore, genetic engineering is increasingly becoming a viable solution for accessing cyanobacterial NPs from native producers, which we discuss below.

#### 3.2.2 Genetic engineering in native producers

**Fig. 3:**
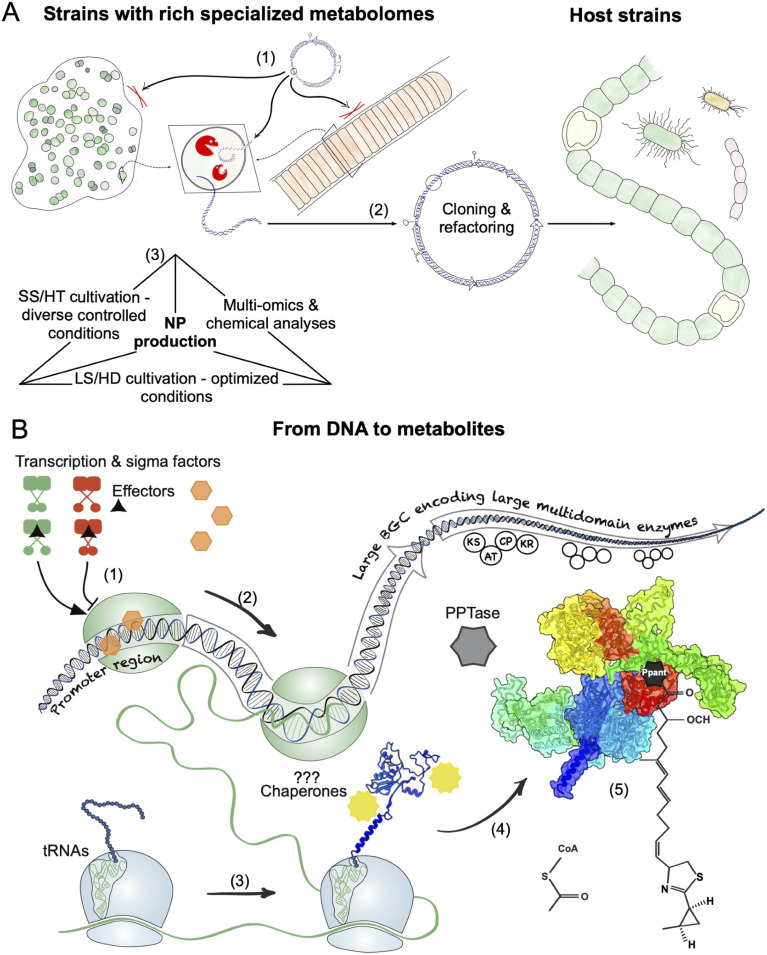
Expression of NP pathways. (A) Heterologous expression pipeline: (1) most cyanobacteria are refractory to genetic manipulations due to physical barriers and cellular defense systems as illustrated in the zoomed-in cross-section of the cells; (2) NP BGCs can be captured into cloning vectors, modified as needed, and the recombinant DNA can be introduced into compatible host strains for heterologous expression; (3) production of NPs in native and heterologous hosts requires a multifaceted approach to better understand NP biology and determine optimal growth conditions. (B) Central Dogma and crucial steps towards NP biosynthesis: (1) the transcription machinery recognizes the promoter sequence and initiates the process; (2) the RNA polymerase transcribes large BGCs and should be highly processive; (3) ribosomes locate an adequate ribosome binding site (RBS) and the start codon to initiate translation, which requires appropriate codon usage and tRNAs; (4) as the enzyme complex is encoded, it undergoes folding, which may require chaperones; (5) the newly encoded enzyme may require post-translational modification/activation by a phosphopantetheinyl transferase (PPTase), and ultimately will participate in NP biosynthesis. SS/HT, small scale/high throughput; LS/HD, large scale/high density.

Genetic studies on native strains can provide important insights to elucidate the regulatory networks and other processes controlling NP biosynthesis, and can activate the expression of BGCs, for example, through the insertion of promoter sequences or the deletion of transcriptional repressors. DNA materials can be introduced into cyanobacteria through natural transformation, electroporation, or conjugation. However, physical barriers such as exopolysaccharides, cell walls, and cellular defense systems (Restriction-Modification systems, CRISPR/Cas) often impede the introduction and maintenance of foreign DNA (Fig. 3). Therefore, genetic manipulations have been conducted in only a few cyanobacterial strains with a rich specialized metabolome. One notable example is *N. punctiforme* PCC 73102, which forms symbiotic associations with plants and is known for its complex life cycle (including the differentiation of heterocysts, hormogonia, and akinetes).^77^ Its genetic tractability has enabled the development of transcriptional reporter and gene knockout strains.^78^ Transcriptional reporter strains showed that HD cultivation led to significant expression changes of its specialized metabolome, leading to the discovery of two cell density-dependent chemical mediators, nostoclides and nostovalerolactones.^72,79^ This strain has also been used to explore the biosynthesis and roles of various secondary metabolites (including scytonemin, microviridins, and nostopeptolides).^78,80,81^

Despite the extensive defense mechanisms of cyanobacteria, innovative approaches can overcome these barriers. For example, foreign DNA can be protected by methylation *in vivo* or *in vitro* prior to its introduction into the strains of interest such as *Anabaena* and *N. punctiforme*.^82^ A few strains of *Arthrospira platensis* (Spirulina), grown industrially for its nutritional value, but refractory to genetic manipulations, gain natural competence for genetic transformation when co-cultured with heterotrophic bacteria. This discovery has enabled the genetic engineering of this organism for biotechnological applications.^83^ Natural competence in *Synechococcus elongatus* is controlled by the circadian clock and is maximal at dusk and early night in darkness.^84^ These findings along with progress in molecular biology and more affordable sequencing and gene synthesis, can substantially help in developing new tools and methods to facilitate genetic manipulation of cyanobacteria, particularly those with multiple predicted BGCs.

#### 3.2.3 *In situ* manipulation (gene editing tools in culture)

Most cyanobacteria live in association with other organisms, making it difficult to isolate axenic cultures. Working with non-axenic cultures complicates the study of these cyanobacteria and their specialized metabolomes. Nonetheless, the ability to study mixed microbial communities grown under native conditions could offer several advantages: it preserves natural community interactions, maintains environmental conditions, and may enable the study in the laboratory of “unculturable” bacteria and possibly enable genetic manipulations of refractory strains such as shown for *A. platensis*.^83^

To study and engineer microbial communities under native environmental conditions, *in situ* manipulation approaches of complex microbial cultures are emerging.^85^ Microbes amenable to genetic manipulation are identified by exposing the microbial community to a randomly integrating mobile genetic element (a transposon). Then the community is sequenced to map and quantify transposon insertions. Once genetically amenable organisms are identified, genetic material can be inserted at a specific locus using approaches that leverage RNA-guided CRISPR-associated transposase systems. Such approaches can be used for the prospection of new genetically tractable strains and opens new avenues for studying cyanobacteria under conditions similar to their native environment.

### 3.3. Cyanobacterial specific challenge #2: improving heterologous expression of cyanobacterial NP pathways

Although there have been numerous cases of successful expression of cyanobacterial NP pathways, there are significant improvements to be made to increase the probability of successful heterologous expression, and increase NP titers. Here we describe the more traditional approaches and then we identify strategies to further enhance these markers of success (Fig. 3).

#### 3.3.1 *Escherichia coli* and other heterotrophic hosts

Fast-growing *E. coli* hosts have been used to express cyanobacterial BGCs. Smaller BGCs with simpler pathways such as RiPPs are generally relatively compatible with the *E. coli* cellular machinery, as evidenced by successful expression of the patellamides, trukamide, anacyclamides, microviridins, and perchlorosins^27^ in addition to the furanolide family of natural products.^86,87^ In contrast, nonribosomal peptides (NRPs) and polyketides (PKs) often have larger, more complex BGCs that require coordinated expression of multiple genes. The biosynthesis of NRPs and PKs also requires a suitable phosphopantetheinyl transferase (PPTase), which must be co-expressed with the BGC in the *E. coli* host. Additionally, the folding of large multi-domain enzymes may require specific codon usage, translation rates, and folding chaperones. Finally, some pathways may require co-factors and precursors not natively present in *E. coli*. Despite these challenges, *E. coli* has been used to express various cyanobacterial NRPs and PKs, such as hapalosin,^88^ anabaenopeptins,^89^ lyngbyatoxin A,^90^ mycosporine-like amino acids (MAAs),^91^ radiosumins,^92^ and microcystins.^27,93^ Regardless of which class of BGC is selected for expression, native cyanobacterial promoters often need to be refactored for compatibility with *E. coli*. Other heterotrophic organisms like *Streptomyces venezuelae* and *Saccharomyces cerevisiae* have also been explored for expressing some of these compounds.^94,95^ To further improve compatibility, phylogenetically related organisms may serve as more suitable hosts for expressing cyanobacterial NPs.

#### 3.3.2 Traditional cyanobacterial genetic models

A few model cyanobacteria, including *S. elongatus* PCC 7942, *Synechococcus* PCC 7002, *Synechocystis* PCC 6803, and *Anabaena* PCC 7120, have been used to produce terpenes, alcohols, carboxylic acids, and free fatty acids.^96,97^*S. elongatus* PCC 7942 was also engineered with the PKS-based mycocerosic pathway to produce multimethyl-branched esters (MBEs)^98^ but failed to produce more complex NRP/PK compounds.^99,100^*Synechocystis* PCC 6803 has been used to produce MAAs like shinorine.^101^ For complex NRPS/PKS-type BGCs, *Anabaena* PCC 7120 appears to be a more suitable host due to its promiscuous Sfp-type PPTase and the compatibility of its cellular machinery with many cyanobacterial promoters. It has been used to produce various compounds, including lyngbyatoxin A, pendolmycin, teleocidins, cryptomaldamide, columbamides, and mycosporine.^27^ Despite these successes, these strains often exhibit slow growth rates compared to heterotrophic hosts. Additionally, other factors, for example the availability of precursors or the pathway's potential toxicity may vary between host strains and emphasize the need for additional hosts for the heterologous expression of NP pathways.

#### 3.3.3 Less studied cyanobacterial strains and recent isolates

In recent years, several fast-growing and stress tolerant *Synechococcus* strains have emerged, such as UTEX 2973, PCC 11801, PCC 11802, and PCC 11901.^102–104^ Under high light and optimum temperature conditions, the growth rates of these strains are comparable to *S. cerevisiae*, with UTEX 2973 achieving a doubling time of about two hours. UTEX 2973 has been used to produce hapalindole alkaloids from the refactored cluster of *Fischerella ambigua*.^105^ Despite its fast-growing phenotype, UTEX 2973 shares the same limitations as its closely related strain, PCC 7942, such as low salt-tolerance, a small genome, and limited metabolic capabilities, which may hinder the production of certain compounds. PCC 11801 and PCC 11802 are also closely related to PCC 7942 but are tolerant to high levels of NaCl. In contrast, PCC 11901 is more closely related to PCC 7002 and phylogenetically distant from PCC 7942. It can grow in a wide range of salinities and accumulate significantly more biomass than other strains. Genetic tools are increasingly available for these strains, but further studies are needed to assess their suitability for expressing NP pathways.^106^

Only about a dozen other strains of cyanobacteria have been reported to be genetically tractable.^107^ The filamentous strain *Leptolyngbya* BL0902 is suitable for large-scale cultivation. *Anabaena* ATCC 33047 shows rapid growth, nitrogen fixation, and high salt tolerance, making it a promising alternative to PCC 7120. The symbiotic strain *Nostoc punctiforme* PCC 73102 harbors several BGCs, which may help understand the regulation mechanisms for specialized metabolites and provide clues to improve the expression of BGCs.

#### 3.3.4 New host strains

To maximize the production of new NPs, candidate host strains should grow rapidly, have broad metabolic capabilities, and have a transcription and a translation machinery (*e.g.* sigma/transcription factors, chaperones) compatible with heterologous BGCs including their promoters, ribosome binding sites, and codon usage. Additionally, resistance to various stresses, the ability to grow in a wide range of salinities, and to reach high cell density are important factors to consider. Evidently, the new host strains must be amenable to genetic modifications using recombinant DNA techniques. For example, new strains should support broad-host-range plasmids and genomic integration by homologous recombination and allow for unmarked mutants using negative selection markers or CRISPR/Cas systems. Other factors that help genetic engineering, such as the ability to grow on agar plates, form isolated colonies, and sensitivity to antibiotics for selection are also important determinants for new host strains. As discussed above, bioprospection efforts and innovative approaches will enhance the discovery and development of new host strains, including strains that are currently refractory to genetic manipulations.

#### 3.3.5 Improving growth rate and compound titer

Metabolic engineering and directed evolution can be deployed to improve the growth rate and compound titer of the host.

Typically, genetic engineering in cyanobacteria has relied on antibiotic markers and the selection of recombinant clones under antibiotic pressure. For more advanced engineering, several approaches have been developed to construct markerless mutant strains. Recent methods rely on CRISPR/Cas systems. Cas9 and Cas12a have been successfully used in various cyanobacterial strains, with Cas12a being preferred due to its minimal impact on cell growth.^108,109^ Additionally, the Cas12a system allows for multiple guide RNAs to be expressed from a single array, facilitating the editing of multiple loci simultaneously. For metabolic engineering, CRISPR systems greatly simplify the construction of complex strains that would otherwise require multiple antibiotic genes and enable genetic modifications in native loci without polar effects, including small modifications. CRISPR interference (CRISPRi) technologies, using inactivated Cas nucleases, have also been developed to regulate gene expression at transcriptional and translational levels.^108^

Moreover, metabolic engineering for improved growth and increased compound yield can benefit from Genome-Scale Models (GSMs). GSMs can predict metabolome and flux changes to identify potential targets for higher production and have been developed for a few model strains like *S. elongatus* PCC 7942, UTEX 2973 and *Synechocystis* PCC 6803.^107^ Tools like OptFlux and OptForce help identify engineering targets to improve specific metabolite production, such as *n*-butanol in PCC 6803.^110^ These useful models are continuously being improved to detect key metabolic differences between strains and identify bottlenecks to improve growth and compound production.

To improve fitness and productivity, Adaptive Laboratory Evolution (ALE) experiments can be deployed. This involves selecting mutant strains that outperform the original population through repeated culture transfers under selective conditions or various stresses. ALE experiments, generally accelerated by mutagenic agents or hypermutation systems, have been used in *Synechocystis* PCC 6803 and *S. elongatus* PCC 7942 and resulted in strains with better tolerance to high light, temperature, and salt stress, as well as strains with increased production of phenylpropanoids or better ethanol tolerance.^111–114^

### 3.4. Cyanobacterial specific challenge #3: increasing our fundamental understanding of cyanobacterial physiology, biosynthesis, and regulation to help guide host development

Environmental conditions including abiotic factors, discussed above, and biotic factors can impact growth and the regulation of pathways encoding for the specialized metabolomes. Better knowledge of the specific conditions that induce expression of different NP BGCs and a deeper understanding of the regulation mechanisms that govern NP pathways can be used in the laboratory to elicit the production of NPs (Fig. 3). However, while the regulation of gene expression in cyanobacteria has been extensively studied for fundamental processes like photosynthesis and nitrogen fixation, research on the regulation of specialized metabolomes in cyanobacteria is limited and largely focused on freshwater toxin pathways.^71,115^ Moreover, beside physical and chemical parameters, biotic factors and the endogenous circadian clock may affect the regulation of the specialized metabolome but little is known about their effect on the production of NPs.

In natural habitats, cyanobacteria live in association or compete with various microorganisms. These interactions affect the growth of cyanobacteria and likely their secondary metabolism. Several NPs have antimicrobial properties,^116^ while others could mediate communication between cyanobacteria and their symbiotic partners.^117^ However, the impact of other organisms on cyanobacterial metabolism is largely unknown. Different studies have shown varied effects on the specialized metabolome. The co-culture of heterotrophic bacteria with *Microcystis* led to degradation of compounds released in the media while negligible intracellular changes were found.^118^ In contrast, significant differences in the specialized metabolome of *N. punctiforme* PCC 73102 were found when the strain was grown associated with its host in comparison to free-living.^81^

Cyanobacteria rely heavily on light for their metabolism and have an endogenous circadian clock to predict day/night cycles and adjust expression of most genes, accordingly.^119,120^ The cyanobacterial clock has been extensively studied in *S. elongatus* PCC 7942 and functional clocks have also been described in *Anabaena* PCC 7120, *Cyanothece* ATCC 51142, and *Synechocystis* PCC 6803.^121–123^ Despite the pervasive control of gene expression by the clock in cyanobacteria, only a very few studies have investigated how day/night cycles may affect specialized metabolomes and have led to mixed results.^124^ Interestingly, the production of different jamaicamides in *Moorena producens* (formerly *Lyngbya majuscula*) occurs at different times of the day,^24^ suggesting that it could be regulated by the clock. In addition to collecting samples at different times of day or growing cultures under different light regimens, genetic approaches that manipulate the clock could be used to maximize yields.^125^

High-throughput methods to grow and monitor cultures under controlled conditions could help assess more comprehensively the role of abiotic and biotic factors, as well as endogenous mechanisms (such as the circadian clock) governing BGC expression.

#### 3.4.1 Transcriptional level

Understanding the mechanisms controlling BGC expression is essential to unlock the potential of cyanobacterial NPs, whether using native producers or heterologous hosts. A few NP pathway promoters have been identified and characterized mostly for major freshwater toxin pathways.^115^ Potential NtcA binding sites have been repeatedly identified and possibly link toxin production to nitrogen assimilation and carbon metabolism.^71^ High-throughput promoter-reported transcriptional fusion studies in *N. punctiforme* PCC 73102 revealed new pathways and cell-density-dependent chemical mediators.^79^ However these studies represent a very small number of BGCs/promoters compared to the diversity of cyanobacterial specialized metabolomes.

For successful heterologous expression, the host machinery and BGC promoters must be compatible. This is achievable through pathway refactoring or promoter swapping. Several inducible and constitutive promoters from cyanobacteria and *E. coli*, as well as synthetic promoters^126^ and fully orthogonal systems, using the T7 RNA polymerase and its cognate promoter, have been characterized in several strains of cyanobacteria.^98^ While refactoring BGCs and exchanging promoters has been effective for NP biosynthesis in *E. coli*, *S. elongatus* and *Anabaena* PCC 7120, these strategies become challenging with large BGCs.^89,105,127^ Alternatively, transforming a BGC with its native regulatory sequences into a compatible host is feasible. *Anabaena* PCC 7120 appears to be a suitable host for several unmodified cyanobacterial BGCs.^99,100,128,129^ Additionally, several other BGC promoters from marine cyanobacteria have appeared compatible with its transcription machinery.^128^ An interesting approach has been to evaluate whether the 12 sigma factors of *Anabaena* PCC 7120 could enable the expression of cyanobacterial promoters in *E. coli*. Although no individual sigma factor could drive the expression of an entire BGC, several sigma factors could drive the expression of individual promoters.^130^

Combining genetic approaches with controlled culture methods for a better understanding of the specialized metabolome regulatory networks can help develop broadly applicable approaches for NP expression in both native and heterologous hosts.

#### 3.4.2 Translational and post-translational levels

NP biosynthesis often depends on large multidomain proteins. Their expression as functional enzymes may pose several challenges. Common issues in protein expression, from RNA stability to potential protein toxicity, are likely to be amplified with the protein size. The complexity of large multidomain proteins increases the likelihood of misfolding, leading to non-functional or aggregated proteins. Different domains may fold independently at different rates increasing the risk of aggregation before complete folding. The expression of large proteins also places a greater burden on cellular resources, and they are more likely to interfere with cellular processes due to their multiple domains and physical size. While native organisms have evolved to properly fold and stabilize their proteins, expression of such proteins in a heterologous host may require several adjustments. These may include fine tuning of expression levels, using modified culture conditions, optimizing codon usage, and expressing additional tRNA and chaperones.^131^

#### 3.4.3 Trade-offs between growth, storage and production of NPs

The heterologous expression of NP pathways in cyanobacteria requires balancing growth, storage, and NP biosynthesis. Although this area requires further research, a few strategies can help address these challenges. For example, using inducible promoters or stationary phase-induced promoters can provide temporal control over NP production, driving BGC expression during periods of reduced growth. Additionally, as previously mentioned, GSM models can predict metabolic fluxes and identify targets to redirect metabolic fluxes towards NP biosynthesis while limiting the burden on cell growth and maintenance.

## 4. Conclusions

A common theme among cyanobacterial natural products is their biological potency, target diversity and opportunity to discover new mechanisms of action. Their structures can lead to first-in-class or best-in-class drug candidates or at least serve as tool compounds to discover new cell biology. The application of synthetic biology has lagged behind in cyanobacteria compared to Actinobacteria (particularly *Streptomyces*) and Pseudomonadota, but with new tools such as CRISPR and a focus on a sustainable green bioeconomy, this is poised to change. Here we have outlined some challenges and some of the resources needed to push forward the synthetic biology of cyanobacteria with a special emphasis on NRPS, PKS, and NRPS–PKS derived natural products/specialized metabolites. To advance synthetic biology in cyanobacteria and thus accelerate drug discovery and green chemistry efforts, we recommend a greater investment in the study of their BGCs, investigation of more cyanobacterial strains, establishment of an open data sharing platform that includes both positive and negative results, and the creation of collaborative research teams or centers.

## 6. Conflicts of interest

There are no conflicts of interest to declare.

## Data Availability

The data and analytical pipeline used in the generation of Fig. 2 is available at our GitHub repository https://github.com/dtdoering/2025-cyano_bgc_perspective.
